# Heterogeneous Associations Between Pandemic-Induced Socioeconomic Hardships and COVID-19 Vaccine Uptake by Sexual Orientation and Gender Identity: A Nationally Representative Analysis in the United States

**DOI:** 10.3390/vaccines12111277

**Published:** 2024-11-13

**Authors:** JungHo Park, Byoungjun Kim

**Affiliations:** 1 Department of Housing & Interior Design (BK21 Four AgeTech-Service Convergence), Kyung Hee University College of Human Ecology, Seoul 02447, Republic of Korea; 2 Department of Surgery, New York University Grossman School of Medicine, New York, NY 10016, USA; 3 Department of Population Health, New York University Grossman School of Medicine, New York, NY 10016, USA

**Keywords:** COVID-19 pandemic, vaccine uptake, sexual orientation, gender identity, pandemic-induced socioeconomic hardships

## Abstract

**Background/Objectives:** Socioeconomic hardship during the COVID-19 pandemic was associated with lower vaccine uptake. Since the pandemic has exacerbated socioeconomic challenges faced by sexual and gender minority populations, including employment income loss, housing instability, food insufficiency, and household expense difficulty, this study investigated the disparities in COVID-19 vaccine uptake among these populations. **Methods:** Using the U.S. Census Bureau Household Pulse Survey, a nationally representative sample of 1,767,966 individuals (6% gay or lesbian, 4.2% bisexual, 1.6% something else, and 90.6% heterosexual respondents), we quantified the COVID-19 vaccine uptakes among sexual and gender minorities, as well as the effect measure modifications by socioeconomic hardships. **Results:** Despite higher vaccine uptake rates among sexual and gender minorities compared to their heterosexual counterparts, socioeconomic hardships triggered by the pandemic among these populations were associated with decreased vaccine uptake. Importantly, the effect measure modifications by socioeconomic hardships were more pronounced among sexual and gender minority status compared to heterosexual individuals. **Conclusions:** These results highlight the critical need to address socioeconomic hardships among sexual and gender minorities to enhance vaccine uptake, along with the pre-existing and exacerbated social and economic disadvantages during the COVID-19 pandemic.

## 1. Introduction

Since the onset of the COVID-19 pandemic, LGBTQ (lesbian, gay, bisexual, transgender, and queer) communities in the United States have borne the brunt of COVID-19 infection risk, morbidity, and mortality [1,2]. The elevated prevalence of pandemic risk within LGBTQ populations is exacerbated by a wide range of pre-existing and persistent socioeconomic obstacles and inequalities that disadvantage sexual and gender minorities [3]. Even before the COVID-19 pandemic, LGBTQ communities reported significantly poorer access to and quality of healthcare (e.g., lack of insurance and medical mistrust due to discrimination), as well as higher rates of poverty, unemployment, inadequate access to nutritious food, housing instability, and difficulties in covering household expenses compared to their non-LGBTQ counterparts [1,4,5,6]. In the middle of the COVID-19 pandemic, LGBTQ individuals faced heightened levels of job loss, financial instability, unmet basic needs, social isolation, and impediments to accessing healthcare [2,5,7]. The persisting and acute hardships due to the pandemic highlight an urgent call for further studies and policy responses, which is particularly true when these factors are identified to negatively impact health-related activities and may affect COVID-19 vaccine uptake within LGBTQ societies [3,4,8].

Despite experiencing greater socioeconomic hardships, studies indicate that LGBTQ individuals exhibit higher levels of interest and confidence in COVID-19 vaccines compared to their non-LGBTQ counterparts [9,10]. The U.S. Centers for Disease Control and Prevention (CDC) reported that lesbian, gay, and bisexual individuals in the U.S. have greater confidence in the safety of COVID-19 vaccines and are more likely to get vaccinated than heterosexuals [11]. Similar results were reported in that there are higher vaccine confidence and vaccination rates among lesbian, gay, and bisexual persons relative to their counterparts [12,13,14].

Despite the high levels of vaccination and trust in vaccines among LGBTQ communities, unique obstacles to COVID-19 vaccine uptake for sexual and gender minorities persist. According to the Theory of Planned Behavior, an individual’s intention to get vaccinated is likely influenced by their socioeconomic hardships [15]. Vaccination intentions can also be affected by social and demographic factors. Despite the ongoing and disproportionate socioeconomic hardships since the beginning of the COVID-19 pandemic in early 2021, there has been little attention given to LGBTQ individuals from the perspectives of pandemic surveillance and research [15,16]. By September 2024, over three and a half years after the initial COVID-19 confirmed case, public reports and statistics were available on the impact of the COVID-19 pandemic-induced hardships on vaccine uptake among LGBTQ communities [15,17,18]. Given the greater prevalence of socioeconomic hardship, barriers to healthcare access, and the lack of data and statistics on sexual and gender minorities during the COVID-19 pandemic, understanding the potential modifying role of sexual and gender identity in the relationship between pandemic-induced hardships and COVID-19 vaccine uptake is of importance [15,19,20].

We seek to offer a better understanding of the landscape of COVID-19 vaccination by analyzing both between- and intra-group differences across sexual orientations and gender identities. Utilizing a national representative survey of U.S. adults, the study aims to (1) estimate the rate of COVID-19 vaccine uptake by sexual orientation and gender identity; (2) assess differences in COVID-19 vaccination based on various socioeconomic hardships due to the pandemic, including employment income loss, food insufficiency, housing instability, and household expense difficulties, as well as sociodemographic factors across sexual orientations and gender identities; and (3) determine if effect measure modification occurs in the association between pandemic-induced socioeconomic hardships and COVID-19 vaccine uptake depending on sexual orientation and gender identity.

## 2. Data and Methods

### 2.1. U.S. Census Bureau’s Household Pulse Survey Public Use File

Data on COVID-19 vaccination among LGBTQ individuals remain scarce, primarily due to the relative lack of systematic data on sexual and gender minorities at both federal, state, and local levels. The Household Pulse Survey (HPS), administered by the U.S. Census Bureau and the National Center for Health Statistics (NCHS) in partnership with other significant federal agencies, is a nationally representative survey designed to evaluate the social, economic, and health-related difficulties driven by the COVID-19 pandemic on adult general households in the U.S. This research analyzed data from a full three-year period of the HPS (from 21 July 2021 to 22 July 2024—corresponding to Phase 3.2 Week 34 to Phase 4.1 Cycle 7 according to U.S. Census Bureau terminology) during which new survey questions on sexual orientation and gender identity were introduced and made available for analysis. This study employed the Public Use File of the HPS, a publicly accessible microdata set that includes responses from individual survey participants. We extracted a nationally representative sample of 1,767,966 respondents from different sexual orientations and gender identities who answered all the questions without any missing data. The sample size of raw data from the Household Pulse Survey microdata is shown in Supplemental Table S1.

### 2.2. Measures

#### 2.2.1. Outcome: COVID-19 Vaccine Uptake

We utilized the question “Have you ever received a COVID-19 vaccine?” with binary response options of (1) yes or (2) no. This question was designed to determine whether a respondent had received at least one dose of a COVID-19 vaccine or booster, irrespective of the manufacturer. Given the full three-year analysis period of this study (21 July 2021–22 July 2024) during the COVID-19 pandemic, this information may help to identify consistently vaccine-hesitant subpopulation groups across different sexual orientations and gender identities. The detailed survey questionnaire and corresponding answer options are shown in Supplemental Table S2.

#### 2.2.2. Effect Measure Modifier: Socioeconomic Hardships Posed by the COVID-19 Pandemic

A broad spectrum of social and economic hardship experienced during the COVID-19 pandemic may influence vaccine uptake. These hardships were quantified by four individual-level binary measures (yes = 1; no = 0): (1) employment income loss, (2) housing instability, (3) food insufficiency, and (4) household expense difficulty. We assessed all four variables in a single model. Employment income loss was assessed based on whether the respondent or anyone in their household experienced a loss of employment income during the pandemic. Instable status of housing cost payment was determined by whether the respondent indicated that they did not pay their past month’s mortgage or rent on time. Food insufficiency was measured by whether the respondent reported not having enough of the types of food they wanted to eat over the past seven days. Household expense difficulty was identified when a respondent reported having difficulty paying for usual household expenses, which could include food, mortgage or rent, car expenses, medical payments, student loans, and other similar expenses.

#### 2.2.3. Sexual Orientation and Gender Identity

The sexual orientation of respondents was identified through the question “Which of the following best represents how you think of yourself?”, with the following response options: (1) Straight, (2) Gay or lesbian, (3) Bisexual, (4) Something else, and (5) I don’t know/nonresponse/missing. A categorical variable for sexual orientation was created, using straight as the reference group and excluding respondents who selected option 5.

Gender identity was determined by responses to the question “Do you currently describe yourself as male, female, or transgender?” The response options were as follows: (1) Male, (2) Female, (3) Transgender, (4) None of these/nonbinary, and (5) nonresponse/missing. A categorical variable was constructed to specify gender identity, with male as the reference group, excluding respondents who selected nonresponse/missing.

#### 2.2.4. Household and Individual-Level Characteristics

Household and individual-level characteristics were all constructed from the Household Pulse Survey microdata, encompassing demographic covariates, socioeconomic statuses, and health insurance status. Demographic covariates include age group, race and ethnicity, marital status, size of household, and the count of children. Social and economic statuses encompass educational attainment, employment status, household income, homeownership, and the type of housing structure. To account for health-related covariates, health insurance status and two self-reported measures of mental health—anxiety and depression—were also considered.

#### 2.2.5. Spatial and Temporal Fixed Effects

To control for spatiotemporal differences in COVID-19 preventive behaviors and vaccine uptake during the pandemic, a set of survey week dummy variables ranging from survey week 1 to week 37 were included across models. Additionally, a binary variable indicating residency within one of the fifteen most populous metropolitan statistical areas (MSAs) was included to account for the metropolitan effect on vaccine uptake. The MSAs include New York, Los Angeles, Chicago, Dallas, Houston, Washington, D.C., Miami, Philadelphia, Atlanta, Phoenix, Boston, San Francisco, Riverside, Detroit, and Seattle.

### 2.3. Statistical Analyses

All statistical analyses were performed by using Stata/MP 18 software (StataCorp, College Station, TX, USA) [21,22]. To estimate the effect modification by sexual orientation and gender identity in the associations between pandemic-induced socioeconomic hardships and COVID-19 vaccine uptake, we employed multilevel mixed-effects logistic regression models within a random intercept framework, adjusting for individual and household-level variables (melogit in Stata/MP 18). A single interaction term between sexual orientation (or gender identity) and one of the four types of pandemic-induced hardships—employment income loss, housing instability, food insufficiency, and household expense difficulty—was included in the model at a time. Given that the state-level error terms may be correlated among samples within an identical state, we utilized standard errors clustered at the state level to partially address heteroscedasticity.

## 3. Results

### 3.1. Descriptive Statistics by Sexual Orientation

Table 1 shows the descriptive statistics on the prevalence of COVID-19 vaccine uptake by sexual orientations such as straight, gay or lesbian, bisexual, and something else. Among straight respondents, the largest subgroup was female (56.8%), non-Hispanic White (77.1%), aged 65–74 years (21.6%), married (59.9%), no child (67.9%), two-person household (39.6%), had a BA or higher education (56.3%), an annual household income of USD 100,000–USD 149,999 (18.8%), not working (39.8%), had private insurance (55.3%), no anxiety (78.9%), no depression (84.5%), and lived in a non-metropolitan area (70.1%). Additionally, 8.9% experienced employment income loss, 4.3% faced housing instability, 29.6% had food insufficiency, and over half (51.6%) encountered household expense difficulties. The overall rate of COVID-19 vaccine uptake among straight individuals was 88.6%, with the highest rates among those who had no employment income loss (89.3%), no housing instability (89.0%), no food insufficiency (92.0%), no household expense difficulty (93.9%), male (88.9%), non-Hispanic Asian and Pacific Islander (97.2%), aged 75 or older (96.0%), married (89.7%), no child (90.9%), two-person household (91.4%), had a BA or higher education (94.1%), an annual household income of USD 150,000 or higher (95.1%), worked for a non-profit (94.1%), had both public and private health insurance (92.0%), no anxiety (89.5%), no depression (89.4%), and resided in a metropolitan area (92.5%).

Gay or lesbian respondents were predominantly male (59.6%), non-Hispanic White (76.7%), aged 35–44 (21.1%) or 55–64 years (21.9%), unmarried (65.1%), no child (88.3%), two-person household (47.0%), had a BA or higher education (65.3%), an annual household income of USD 150,000 or higher (23.5%), worked for a private company (38.1%), had private insurance (64.4%), no anxiety (70.1%), no depression (77.1%), and lived in a non-metropolitan area (59.4%). Approximately 10.3% experienced employment income loss, 4.4% faced housing instability, 28.7% had food insufficiency, and 49.5% encountered household expense difficulties. The overall prevalence of COVID-19 vaccination among gay or lesbian respondents was 96.3%, with the highest rates among subgroups with no employment income loss (96.7%), no housing instability (96.6%), no food insufficiency (98.0%), no household expense difficulty (98.5%), male (97.4%), Asian and Pacific Islander (99.1%), aged 75 or older (98.6%), married (97.5%), no child (96.9%), two-person household (97.2%), had a BA or higher education (98.6%), an annual household income of USD 150,000 or higher (99.1%), worked for a non-profit (98.8%), had private health insurance (97.4%), and lived in a metropolitan area (97.9%).

Bisexual and Something else respondents shared similar characteristics. The largest subgroups were non-Hispanic White (76.2% and 70.8%, respectively), aged 25–34 years (36.2%; 29.1%), unmarried (62.1%; 66.0%), no child (68.9%; 73.6%), two-person household (38.1%; 35.2%), had a BA or higher education (54.2%; 55.6%), an annual household income of USD 25,000–USD 49,999 (23.9%; 25.3%), worked for a private company (42.4%; 37.3%), had private insurance (65.0%; 59.7%), and lived in a non-metropolitan area (70.2%; 69.8%). Among gender identities, 70.2% and 22.7% of bisexual individuals were female and male, respectively; 51.3% and 24.5% of Something else respondents were female and male, respectively, with 8.4% transgender and 16.0% nonbinary (None of these respondents). About 13.6%, 6.6%, 42.9%, and 66.2% of bisexual individuals experienced employment income loss, housing instability, food insufficiency, and household expense difficulty, respectively. Similarly, 16.0%, 6.3%, 46.0%, and 67.4% of Something else respondents faced these individual hardships. The overall rates of COVID-19 vaccination were 91.4% for bisexual individuals and 90.6% for Something else respondents, with the highest rates among those with no employment income loss (92.3% for bisexual; 91.7% for Something else), no housing instability (92.2%; 91.1%), no food insufficiency (95.4%; 94.1%), no household expense difficulty (96.5%; 94.4%), Asian and Pacific Islander (97.7%; 94.9%), no child (94.1%; 92.9%), two-person household (94.0%; 93.2%), had a BA or higher education (97.4%; 94.6%), worked for a non-profit (97.5%; 96.9%), had private health insurance (95.2%; 94.5%), and lived in a metropolitan area (94.4%; 93.4%). For gender identity, the highest vaccine uptake was among transgender (95.2%) and None of these (96.1%) for bisexual respondents and female (92.4%) and transgender (92.3%) for Something else respondents.

### 3.2. Multilevel Mixed-Effects Logistic Regression Results

Table 2 presents the multilevel mixed-effects logistic regression results on the associations between pandemic-induced socioeconomic hardships and COVID-19 vaccine uptake, adjusting for the aforementioned individual- and household-level covariates. The analysis included a nationally representative sample of 1,767,966 respondents across sexual orientations and gender identities.

Adults who experienced employment income loss showed a lower rate of COVID-19 vaccine uptake (OR = 0.93, 95% CI = 0.91, 0.95) compared to those without income loss. Similarly, respondents who faced housing instability (OR = 0.85, 95% CI = 0.84, 0.87) were less likely to be vaccinated compared to those who paid their rent or mortgage on time. Those who experienced food insufficiency (OR = 0.73, 95% CI = 0.72, 0.75) and household expense difficulty (OR = 0.73, 95% CI = 0.72, 0.75) also reported a lower rate of vaccine uptake relative to those without these hardships.

The odds ratios for vaccine uptake were higher among gay or lesbian (OR = 3.17, 95% CI = 2.97, 3.39), bisexual (OR = 2.23, 95% CI = 2.15, 2.32), and Something else respondents (OR = 1.79, 95% CI = 1.68, 1.91) compared to straight respondents. Females (OR = 1.11, 95% CI = 1.10, 1.13) and transgender individuals (OR = 1.82, 95% CI = 1.60, 2.09), as well as None of these respondents (OR = 1.08, 95% CI = 1.03, 1.16), had higher odds of receiving the vaccine compared to males. Non-Hispanic Asians and Pacific Islanders (OR = 3.65, 95% CI = 3.29, 4.05), Hispanics (OR = 1.62, 95% CI = 1.48, 1.78), and non-Hispanic Blacks (OR = 1.56, 95% CI = 1.39, 1.75) had higher odds of vaccination compared to non-Hispanic Whites.

Individuals aged 75 or older (OR = 4.48, 95% CI = 4.09, 4.93) and those in middle-aged groups had higher rates of COVID-19 vaccine uptake compared to the youngest age group (18–24 years). Married individuals (OR = 1.01, 95% CI = 0.99, 1.04) were more likely to be vaccinated than unmarried respondents, while those with one or more children in the household (OR = 0.81, 95% CI = 0.80, 0.83) had a lower rate of vaccine uptake compared to no-child households. Compared to single-person households, those in six-or-more-person households (OR = 0.57, 95% CI = 0.55, 0.61) and two- to five-person households had lower odds of vaccination. Higher education levels were associated with higher odds of vaccination, with high school diploma holders (OR = 1.08, 95% CI = 1.02, 1.16), those with some college or an associate’s degree (OR = 1.50, 95% CI = 1.40, 1.62), and those with a bachelor’s degree or higher (OR = 3.20, 95% CI = 2.96, 3.47) all having higher odds compared to those with less than a high school education. Higher odds of vaccination were observed among respondents with a household income of USD 150,000 or more (OR = 2.11, 95% CI = 1.99, 2.24) and those in middle-income groups compared to those with an income of USD 25,000 or less. Government employees (OR = 1.33, 95% CI = 1.28, 1.39) and non-profit workers (OR = 1.79, 95% CI = 1.71, 1.89) had higher odds of vaccination compared to those who were not working, while others who were self-employed or worked for a family business (OR = 0.66, 95% CI = 0.64, 0.69) had lower odds; private company workers did not significantly differ from non-workers. Private insurance holders (OR = 1.35, 95% CI = 1.33, 1.38) and private/public insurance holders (OR = 1.24, CI = 1.23, 1.27) are more likely to get vaccinated than public insurance holders while individuals without health insurance (OR= 0.75, 95% CI = 0.73, 0.78) are less likely to receive vaccines. People with symptoms of anxiety (OR = 1.23, 95% CI = 1.22, 1.26) and depression (OR = 1.05, 95% CI = 1.03, 1.07) are more likely to receive a COVID-19 vaccine than individuals without these mental health problems. Additionally, residents of metropolitan areas (OR = 1.23, 95% CI = 1.22, 1.26) had higher odds of receiving the COVID-19 vaccine compared to non-metropolitan rural residents.

### 3.3. Effect Measure Modification by COVID-19 Pandemic-Induced Socioeconomic Hardships

**Figure 1:**
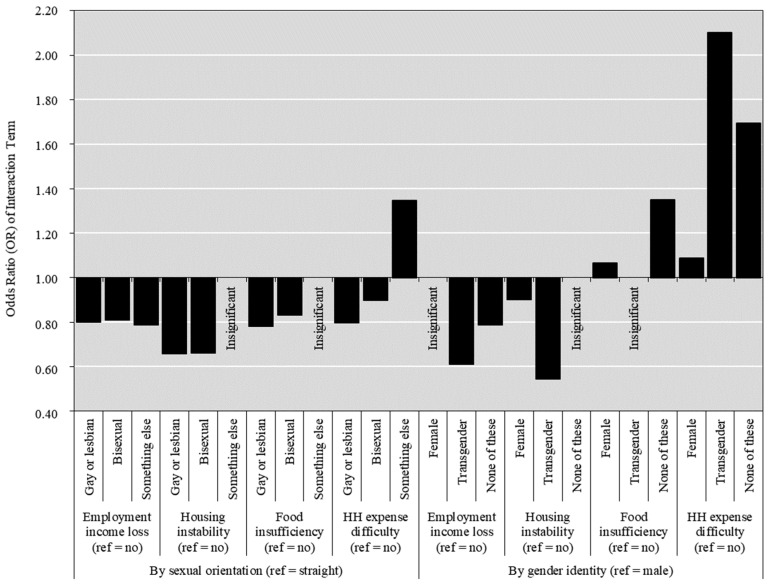
Odds ratio of interaction terms between COVID-19 pandemic-induced socioeconomic hardships and sexual orientation and gender identity, U.S. Household Pulse Survey, 21 July 2021—22 July 2024 (*N* = 1,767,966).

Table 3 presents the summarized results of a multilevel mixed-effects logistic regression analysis examining the effect measure modifications by COVID-19 pandemic-induced socioeconomic hardships on the associations of sexual orientation and gender identity with vaccine uptake. We tested one interaction term at a time in eight model estimation results as summarized in Table 3 and visualized in Figure 1. The findings indicate significant effect measure modifications by sexual orientation and gender identity on the associations between various socioeconomic hardships and vaccine uptake. Full model results are provided in Supplemental Tables S3 and S4.

Effect measure modifications by pandemic-induced employment income loss: The analysis revealed a significant effect modification by employment income loss on the association between sexual orientation and COVID-19 vaccine uptake. Specifically, gay or lesbian persons, as well as bisexual respondents who experienced income loss, demonstrated a higher rate of vaccine uptake compared to their straight counterparts. However, this likelihood diminished somewhat for gay or lesbian individuals (OR = 0.80, 95% CI = 0.72, 0.90) and bisexual individuals (OR = 0.81, 95% CI = 0.77, 0.87). Additionally, the interaction between gender identity and employment income loss significantly influenced the likelihood of COVID-19 vaccination. Although transgender individuals and respondents identifying as None of these consistently exhibited the highest rates of vaccination despite income loss, this likelihood notably decreased for transgender individuals (OR = 0.61, 95% CI = 0.50, 0.76) and for None of these respondents (OR = 0.79, 95% CI = 0.70, 0.90).

Effect measure modifications by pandemic-induced housing instability: Instable status of housing cost payment significantly modified the relationship between sexual orientation and the rate of COVID-19 vaccine uptake. Although gay or lesbian individuals and bisexual respondents experiencing housing instability demonstrated the highest probabilities of vaccination, the disparity narrowed for gay or lesbian individuals (OR = 0.66, 95% CI = 0.59, 0.74) and bisexual respondents (OR = 0.66, 95% CI = 0.61, 0.73). Furthermore, housing instability significantly modified the association between gender identity and COVID-19 vaccination likelihood. Despite transgender individuals experiencing housing instability continuing to show the highest vaccination probabilities, the difference in likelihood substantially decreased for transgender individuals (OR = 0.54, 95% CI = 0.38, 0.78).

Effect measure modifications by pandemic-induced food insufficiency: The interaction between sexual orientation and food insufficiency significantly influenced the likelihood of COVID-19 vaccination. Even though gay or lesbian individuals and bisexual respondents with food insufficiency had the highest vaccination probabilities, the gap narrowed for gay or lesbian individuals (OR = 0.78, 95% CI = 0.72, 0.86) and bisexual persons (OR = 0.83, 95% CI = 0.79, 0.88). Food insufficiency also significantly modified the relationship between gender identity and COVID-19 vaccination. Transgender individuals (OR = 1.22, 95% CI = 0.98, 1.54) and those identifying as None of these (OR = 1.35, 95% CI = 1.20, 1.53) who experienced food insufficiency exhibited a higher rate of COVID-19 vaccine uptake compared to their cisgender counterparts.

Effect measure modifications by pandemic-induced household expense difficulty: Household expense difficulty substantially modified the relations between sexual orientation and the rate of COVID-19 vaccine uptake. Gay or lesbian individuals and bisexual respondents experiencing expense difficulty continued to exhibit the highest rates of vaccine uptake, though the disparity narrowed for gay or lesbian individuals (OR = 0.80, 95% CI = 0.72, 0.89) and bisexual individuals (OR = 0.90, 95% CI = 0.85, 0.96). Moreover, household expense difficulty significantly modified the relationship between gender identity and the likelihood of vaccination. Transgender individuals (OR = 2.10, 95% CI = 1.62, 2.73) and those identifying as None of these (OR = 1.69, 95% CI = 1.50, 1.92) experiencing expense difficulty exhibited the highest rates of COVID-19 vaccine uptake.

## 4. Discussion

### 4.1. COVID-19 Public Health Implications

The present study elucidated noteworthy trends regarding the rate of COVID-19 vaccine uptake across different sexual orientations and gender identities. Our multilevel mixed-effect logistic regression analysis revealed findings consistent with previous literature: individuals who experienced employment income loss were less likely to report being vaccinated. Additionally, people in the U.S. facing housing instability, food insufficiency, and household expense difficulties were also less likely to receive the COVID-19 vaccine. These findings highlight a critical area for government intervention, suggesting that financial assistance to cover the cost of vaccination and non-financial support to promote easier access to vaccines should be targeted toward socioeconomically vulnerable and marginalized subpopulations. While non-straight sexual orientation groups exhibited a higher prevalence of vaccination compared to their straight counterparts, the likelihood of vaccination varied among different sexual orientation groups. Specifically, gay or lesbian persons had a higher rate of COVID-19 vaccine uptake compared to bisexual and Something else respondents. These findings align with previous studies that have documented differences in vaccination rates across different sexual orientations and gender identities [3,16,23,24]. Consequently, there may be vaccination disparities across sexual and gender identity groups, underscoring the need to tailor vaccination strategies to address the underlying heterogeneity within sexual and gender minority societies.

A salient finding from our effect measure modification analysis presented that sexual and gender minority groups are more vulnerable and sensitive to pandemic-induced socioeconomic hardship than their counterparts. This study found that gay or lesbian individuals and bisexual respondents with employment income loss—or any form of housing instability, food insufficiency, or household expense difficulty—reported an additionally lowered rate of COVID-19 vaccination compared to straight respondents experiencing similar hardships. Prior research in this area has not yet examined the salient interactions of sexual orientation and gender identity with pandemic-induced socioeconomic hardship, with some studies indicating that individuals with social and economic hardships are less likely to be vaccinated, others showing different rates of COVID-19 vaccine uptake across sexual and gender groups, and some documenting variability in vaccination by sexual orientation, gender identity, and socioeconomic hardship side by side but not in an interconnected manner [6,25,26,27]. This study offers findings of fundamental heterogeneity in COVID-19 vaccination by sexual orientation and gender identity given a variety of socioeconomic hardship, illustrating that sexual and gender minority individuals with socioeconomic hardships may be at the highest risk for avoiding timely vaccination. The evidence of this study may be attributable to more serious mental illnesses such as anxiety and depression [28,29]. Previous studies have highlighted the higher prevalence of socioeconomic hardships among sexual and gender minority groups even before the outbreak of the COVID-19 pandemic. Moreover, individuals facing socioeconomic hardships, especially those identifying as sexual and gender minorities, are more likely to exhibit distrust towards the government, primary care providers, the medical system, and general news media [13,30,31]. They often encounter discrimination and endure systematic obstacles to accessing healthcare, which contributes to lower rates of reported vaccination [2,12,32,33].

### 4.2. Limitations

This study is not without its empirical limitations. Even though the Household Pulse Survey microdata administered by the U.S. Census Bureau provided a large and nationally representative sample of 1,767,966 respondents and offered unique insights into both various pandemic-induced socioeconomic hardships and the rate of COVID-19 vaccine uptake among sexual and gender minorities, the pooled cross-sectional structure of the microdata prevents researchers from testing causal relationships. Further research is also needed to illuminate the role of financial support and addressing obstacles to access to vaccines among subpopulation groups identified by different sexual orientations and gender identities. The availability and scope of HPS data is limited depending on the survey period of analysis, preventing us from controlling for a comprehensive range of individual and household-level factors [34,35,36], community-wide and regional contexts [37,38,39], and federal and local public programs [40,41,42]. Additionally, we did not investigate other types of pandemic-induced hardships, such as experiences of discrimination and social isolation, which may have disproportionately reduced vaccine acceptance among sexual and gender minorities [15,18]. Our findings on lower vaccine uptake among some types of households, including households with one or more children, warrant further research focusing on socioeconomic hardships originating from their household characteristics.

## 5. Conclusions

Our findings highlight the heightened level of COVID-19 pandemic-induced social and economic hardships experienced by sexual and gender minority groups compared to straight individuals. These results emphasize the necessity for targeted financial and non-monetary initiatives aimed at socioeconomically vulnerable populations to directly support costs of vaccination and indirectly improve accessibility to vaccines, with a particular focus on sexual and gender minorities. Furthermore, this study emphasizes the critical modifying role of pandemic-induced socioeconomic hardships on the relationship of sexual orientation and gender identity with COVID-19 vaccine uptake, highlighting the disproportionately lower vaccination rates among sexual and gender minorities. These findings advocate for the need to advance innovative campaigns and strategies to understand and address the myriad sources and causes of socioeconomic hardships among these vulnerable populations. Such vaccination campaigns and strategies are essential to improving vaccination acceptance despite social and economic challenges during future global health crises beyond the COVID-19 pandemic.

## Figures and Tables

**Table 1:** Descriptive statistics of COVID-19 vaccine uptake by sexual orientation, U.S. Household Pulse Survey, 21 July 2021—22 July 2024 (*N* = 1,767,966).

Variables	Straight		Gay or Lesbian		Bisexual		Something Else	
	Total n (%)	Vaccine Uptake n (%)	Total n (%)	Vaccine Uptake n (%)	Total n (%)	Vaccine Uptake n (%)	Total n (%)	Vaccine Uptake n (%)
**Total**	1,601,659 (90.6)	1,417,578 (88.6)	63,852 (3.6)	61,433 (96.3)	73,903 (4.2)	67,518 (91.4)	28,552 (1.7)	25,843 (90.6)
Income loss								
Yes	142,312 (8.9)	115,527 (81.2)	6547 (10.3)	6029 (92.1)	9991 (13.6)	8538 (85.5)	4542 (16)	3834 (84.5)
No	1,459,347 (91.2)	1,302,051 (89.3)	57,305 (89.8)	55,404 (96.7)	63,912 (86.5)	58,980 (92.3)	24,010 (84.1)	22,009 (91.7)
Housing instability								
Yes	67,677 (4.3)	52,498 (77.6)	2808 (4.4)	2480 (88.4)	4806 (6.6)	3814 (79.4)	1773 (6.3)	1457 (82.2)
No	1,533,982 (95.8)	1,365,080 (89)	61,044 (95.7)	58,953 (96.6)	69,097 (93.5)	63,704 (92.2)	26,779 (93.8)	24,386 (91.1)
Food insufficiency								
Yes	472,588 (29.6)	379,448 (80.3)	18,262 (28.7)	16,797 (92)	31,702 (42.9)	27,286 (86.1)	13,123 (46)	11,339 (86.5)
No	1,129,071 (70.5)	1,038,130 (92)	45,590 (71.4)	44,636 (98)	42,201 (57.2)	40,232 (95.4)	15,429 (54.1)	14,504 (94.1)
HH Expense difficulty								
Yes	825,825 (51.6)	689,920 (83.6)	31,590 (49.5)	29,667 (94)	48,896 (66.2)	43,392 (88.8)	19,233 (67.4)	17,049 (88.7)
No	775,834 (48.5)	727,658 (93.8)	32,262 (50.6)	31,766 (98.5)	25,007 (33.9)	24,126 (96.5)	9319 (32.7)	8794 (94.4)
Gender identity								
Male	685,113 (42.8)	608,959 (88.9)	38,010 (59.6)	37,013 (97.4)	16,729 (22.7)	15,599 (93.3)	6989 (24.5)	6179 (88.5)
Female	908,870 (56.8)	802,009 (88.3)	22,822 (35.8)	21,564 (94.5)	51,862 (70.2)	46,840 (90.4)	14,638 (51.3)	13,523 (92.4)
Transgender	401 (0.1)	353 (88.1)	1392 (2.2)	1308 (94)	2561 (3.5)	2438 (95.2)	2374 (8.4)	2191 (92.3)
None of these	7275 (0.5)	6257 (86.1)	1628 (2.6)	1548 (95.1)	2751 (3.8)	2641 (96.1)	4551 (16)	3950 (86.8)
Race/ethnicity								
Non-Hispanic White	1,233,300 (77.1)	1,089,232 (88.4)	48,931 (76.7)	47,361 (96.8)	56,270 (76.2)	51,820 (92.1)	20,195 (70.8)	18,673 (92.5)
Non-Hispanic Black	111,632 (7)	99,014 (88.7)	3589 (5.7)	3229 (90)	3350 (4.6)	2808 (83.9)	1378 (4.9)	1187 (86.2)
Non-Hispanic A&PI	73,382 (4.6)	71,312 (97.2)	2286 (3.6)	2265 (99.1)	2171 (3)	2121 (97.7)	994 (3.5)	943 (94.9)
Non-Hispanic others	57,913 (3.7)	48,022 (83)	2653 (4.2)	2497 (94.2)	4637 (6.3)	4060 (87.6)	2258 (8)	1921 (85.1)
Hispanic	125,432 (7.9)	109,998 (87.7)	6393 (10.1)	6081 (95.2)	7475 (10.2)	6709 (89.8)	3727 (13.1)	3119 (83.7)
Age group								
18–24	19,726 (1.3)	15,206 (77.1)	2051 (3.3)	1901 (92.7)	6263 (8.5)	5616 (89.7)	1786 (6.3)	1590 (89.1)
25–34	164,928 (10.3)	134,880 (81.8)	11,369 (17.9)	10,739 (94.5)	26,697 (36.2)	24,292 (91)	8286 (29.1)	7702 (93)
35–44	296,801 (18.6)	250,671 (84.5)	13,464 (21.1)	12,799 (95.1)	19,591 (26.6)	17,764 (90.7)	7747 (27.2)	7121 (92)
45–54	294,388 (18.4)	253,559 (86.2)	10,756 (16.9)	10,360 (96.4)	9848 (13.4)	8969 (91.1)	4316 (15.2)	3816 (88.5)
55–64	322,038 (20.2)	287,328 (89.3)	13,947 (21.9)	13,557 (97.3)	5941 (8.1)	5543 (93.4)	2977 (10.5)	2630 (88.4)
65–74	345,317 (21.6)	323,854 (93.8)	9526 (15)	9378 (98.5)	4100 (5.6)	3930 (95.9)	2426 (8.5)	2165 (89.3)
75+	158,461 (9.9)	152,080 (96)	2739 (4.3)	2699 (98.6)	1463 (2)	1404 (96)	1014 (3.6)	819 (80.8)
Marital status								
Married	958,241 (59.9)	858,869 (89.7)	22,309 (35)	21,730 (97.5)	28,054 (38)	26,096 (93.1)	9720 (34.1)	8721 (89.8)
Unmarried	643,418 (40.2)	558,709 (86.9)	41,543 (65.1)	39,703 (95.6)	45,849 (62.1)	41,422 (90.4)	18,832 (66)	17,122 (91)
Children in HH								
1+ children	515,448 (32.2)	430,710 (83.6)	7510 (11.8)	6873 (91.6)	23,036 (31.2)	19,683 (85.5)	7566 (26.5)	6352 (84)
No child	1,086,211 (67.9)	986,868 (90.9)	56,342 (88.3)	54,560 (96.9)	50,867 (68.9)	47,835 (94.1)	20,986 (73.6)	19,491 (92.9)
Household size								
Single-person	301,741 (18.9)	273,050 (90.5)	20,371 (32)	19,707 (96.8)	14,447 (19.6)	13,578 (94)	7346 (25.8)	6828 (93)
2-person	632,920 (39.6)	578,273 (91.4)	30,002 (47)	29,143 (97.2)	28,149 (38.1)	26,452 (94)	10,026 (35.2)	9344 (93.2)
3-person	265,714 (16.6)	231,691 (87.2)	7236 (11.4)	6843 (94.6)	13,966 (18.9)	12,635 (90.5)	4774 (16.8)	4350 (91.2)
4-person	232,152 (14.5)	199,657 (86.1)	3717 (5.9)	3502 (94.3)	10,034 (13.6)	8837 (88.1)	3296 (11.6)	2960 (89.9)
5-person	101,834 (6.4)	83,357 (81.9)	1417 (2.3)	1297 (91.6)	4331 (5.9)	3679 (85)	1423 (5)	1236 (86.9)
6 or more persons	67,298 (4.3)	51,550 (76.6)	1109 (1.8)	941 (84.9)	2976 (4.1)	2337 (78.6)	1687 (6)	1125 (66.7)
Education								
Less than high school	26,178 (1.7)	18,878 (72.2)	619 (1)	493 (79.7)	1301 (1.8)	838 (64.5)	912 (3.2)	647 (71)
High school	182,369 (11.4)	140,804 (77.3)	4405 (6.9)	3846 (87.4)	7057 (9.6)	5361 (76)	2546 (9)	2086 (82)
Some college and AA	492,870 (30.8)	411,208 (83.5)	17,192 (27)	16,074 (93.5)	25,505 (34.6)	22,339 (87.6)	9240 (32.4)	8125 (88)
BA+	900,242 (56.3)	846,688 (94.1)	41,636 (65.3)	41,020 (98.6)	40,040 (54.2)	38,980 (97.4)	15,854 (55.6)	14,985 (94.6)
Household income								
Less than USD 25,000	159,865 (10)	125,687 (78.7)	7280 (11.5)	6560 (90.2)	11,281 (15.3)	9168 (81.3)	5360 (18.8)	4446 (83)
USD 25,000–49,999	297,336 (18.6)	250,199 (84.2)	11,820 (18.6)	11,054 (93.6)	17,644 (23.9)	15,533 (88.1)	7198 (25.3)	6450 (89.7)
USD 50,000–74,999	265,677 (16.6)	231,267 (87.1)	10,221 (16.1)	9827 (96.2)	13,449 (18.2)	12,467 (92.7)	5129 (18)	4783 (93.3)
USD 75,000–99,999	227,175 (14.2)	202,226 (89.1)	8428 (13.2)	8206 (97.4)	10,050 (13.6)	9483 (94.4)	3597 (12.6)	3382 (94.1)
USD 100,000–149,999	299,740 (18.8)	273,765 (91.4)	11,161 (17.5)	10,989 (98.5)	11,276 (15.3)	10,875 (96.5)	3837 (13.5)	3630 (94.7)
USD 150,000+	351,866 (22)	334,434 (95.1)	14,942 (23.5)	14,797 (99.1)	10,203 (13.9)	9992 (98)	3431 (12.1)	3152 (91.9)
Work status								
No work	636,450 (39.8)	567,412 (89.2)	19,413 (30.5)	18,494 (95.3)	19,255 (26.1)	16,765 (87.1)	9015 (31.6)	7803 (86.6)
Government	165,095 (10.4)	151,108 (91.6)	7588 (11.9)	7413 (97.7)	8424 (11.4)	8090 (96.1)	3058 (10.8)	2911 (95.2)
Private company	537,832 (33.6)	467,416 (87)	24,325 (38.1)	23,350 (96)	31,312 (42.4)	28,719 (91.8)	10,646 (37.3)	9789 (92)
Non-profit	127,422 (8)	119,850 (94.1)	7194 (11.3)	7107 (98.8)	8461 (11.5)	8248 (97.5)	3116 (11)	3019 (96.9)
Others	134,860 (8.5)	111,792 (82.9)	5332 (8.4)	5069 (95.1)	6451 (8.8)	5696 (88.3)	2717 (9.6)	2321 (85.5)
Health insurance								
Public	276,191 (17.3)	235,568 (85.3)	9868 (15.5)	9267 (94)	12,314 (16.7)	10,101 (82.1)	5257 (18.5)	4556 (86.7)
Private	885,299 (55.3)	792,995 (89.6)	41,078 (64.4)	40,006 (97.4)	47,965 (65)	45,635 (95.2)	17,021 (59.7)	16,077 (94.5)
Both	365,460 (22.9)	336,113 (92)	9515 (15)	9228 (97)	7907 (10.7)	7147 (90.4)	3493 (12.3)	3071 (88)
None	74,709 (4.7)	52,902 (70.9)	3391 (5.4)	2932 (86.5)	5717 (7.8)	4635 (81.1)	2781 (9.8)	2139 (77)
Anxiety								
Yes	339,116 (21.2)	288,056 (85)	19,126 (30)	18,030 (94.3)	35,200 (47.7)	31,767 (90.3)	13,703 (48)	12,351 (90.2)
No	1,262,543 (78.9)	1,129,522 (89.5)	44,726 (70.1)	43,403 (97.1)	38,703 (52.4)	35,751 (92.4)	14,849 (52.1)	13,492 (90.9)
Depression								
Yes	248,713 (15.6)	208,163 (83.7)	14,643 (23)	13,703 (93.6)	26,615 (36.1)	23,718 (89.2)	10,949 (38.4)	9779 (89.4)
No	1,352,946 (84.5)	1,209,415 (89.4)	49,209 (77.1)	47,730 (97)	47,288 (64)	43,800 (92.7)	17,603 (61.7)	16,064 (91.3)
Place of residence								
MSA	479,542 (30)	443,211 (92.5)	25,950 (40.7)	25,381 (97.9)	22,085 (29.9)	20,837 (94.4)	8643 (30.3)	8068 (93.4)
Non-MSA	1,122,117 (70.1)	974,367 (86.9)	37,902 (59.4)	36,052 (95.2)	51,818 (70.2)	46,681 (90.1)	19,909 (69.8)	17,775 (89.3)

Note: Descriptive statistics of survey week are not shown in this table. A&PI = Asian and Pacific Islander. AA = Associate in Arts. BA+ = bachelor’s degree or higher. HH = household. MSA = metropolitan statistical area.

**Table 2:** Multilevel mixed-effects logistic regression results, U.S. Household Pulse Survey, 21 July 2021—22 July 2024 (*N* = 1,767,966).

Variables	OR	(95% CI)	*p*
Employment income loss (ref = no)			
Yes	0.93	(0.91, 0.95)	<0.001
Housing instability (ref = no)			
Yes	0.85	(0.84, 0.87)	<0.001
Food insufficiency (ref = no)			
Yes	0.73	(0.72, 0.75)	<0.001
Household expense difficulty (ref = no)			
Yes	0.73	(0.72, 0.75)	<0.001
Sexual orientation (ref = straight)			
Gay or lesbian	3.17	(2.97, 3.39)	<0.001
Bisexual	2.23	(2.15, 2.32)	<0.001
Something else	1.79	(1.68, 1.91)	<0.001
Gender identity (ref = male)			
Female	1.11	(1.1, 1.13)	<0.001
Transgender	1.82	(1.6, 2.09)	<0.001
None of these	1.08	(1.03, 1.16)	0.01
Race/ethnicity (ref = White)			
Non-Hispanic Black	1.56	(1.39, 1.75)	<0.001
Non-Hispanic A&PI	3.65	(3.29, 4.05)	<0.001
Non-Hispanic others	0.98	(0.91, 1.06)	0.56
Hispanic	1.62	(1.48, 1.78)	<0.001
Age (ref = 18–24)			
25–34	0.85	(0.81, 0.9)	<0.001
35–44	1.05	(0.99, 1.12)	0.12
45–54	1.21	(1.15, 1.28)	<0.001
55–64	1.67	(1.58, 1.78)	<0.001
65–74	3.19	(2.96, 3.45)	<0.001
75+	4.48	(4.09, 4.93)	<0.001
Marital status (ref = unmarried)			
Married	1.01	(0.99, 1.04)	0.32
Children in household (ref = no child)			
One or more children	0.81	(0.8, 0.83)	<0.001
Household size (ref = single person)			
2-person	0.96	(0.95, 0.98)	<0.001
3-person	0.87	(0.85, 0.9)	<0.001
4-person	0.81	(0.79, 0.83)	<0.001
5-person	0.70	(0.68, 0.73)	<0.001
6 or more persons	0.57	(0.55, 0.61)	<0.001
Education (ref = less than High School)			
High school	1.08	(1.02, 1.16)	0.01
Some college and AA	1.50	(1.4, 1.62)	<0.001
BA+	3.20	(2.96, 3.47)	<0.001
HH income (ref = less than USD 25,000)			
USD 25,000–49,999	1.20	(1.18, 1.23)	<0.001
USD 50,000–74,999	1.25	(1.22, 1.28)	<0.001
USD 75,000–99,999	1.32	(1.28, 1.37)	<0.001
USD 100,000–149,999	1.52	(1.47, 1.58)	<0.001
USD 150,000+	2.11	(1.99, 2.24)	<0.001
Work status (ref = no work)			
Government	1.33	(1.28, 1.39)	<0.001
Private company	1.00	(0.98, 1.03)	0.73
Non-profit	1.79	(1.71, 1.89)	<0.001
Others	0.66	(0.64, 0.69)	<0.001
Health insurance (ref = public)			
Private	1.35	(1.33, 1.38)	<0.001
Both	1.24	(1.23, 1.27)	<0.001
None	0.75	(0.73, 0.78)	<0.001
Anxiety (ref = no)			
Yes	1.23	(1.22, 1.26)	<0.001
Depression (ref = no)			
Yes	1.05	(1.03, 1.07)	<0.001
Place of residence (ref = non-MSA)			
MSA	1.23	(1.22, 1.26)	<0.001
Constant	1.99	(1.67, 2.36)	<0.001

Note: OR = odds ratio. CI = confidence interval. Ref = reference group. A&PI = Asian and Pacific Islander. AA = Associate in Arts. BA+ = bachelor’s degree or higher. HH = household. MSA = metropolitan statistical area. All models were adjusted for survey week. Standard errors were clustered at the state level.

**Table 3:** Effect measure modifications by COVID-19 pandemic-induced socioeconomic hardships on the associations of sexual orientation and gender identity with vaccine uptake, U.S. Household Pulse Survey, 21 July 2021–22 July 2024 (*N* = 1,767,966).

Effect Measure Modifications by Pandemic-Induced Employment Income Loss							
Variables	OR	(95% CI)	*p*	Variables	OR	(95% CI)	*p*
Employment income loss (ref = no)				Employment income loss (ref = no)			
Yes	0.95	(0.93, 0.97)	<0.001	Yes	0.94	(0.92, 0.97)	<0.001
Sexual orientation (ref = straight)				Gender identity (ref = male)			
Gay or lesbian	3.30	(3.07, 3.56)	<0.001	Female	1.11	(1.1, 1.13)	<0.001
Bisexual	2.32	(2.23, 2.42)	<0.001	Transgender	2.08	(1.8, 2.4)	<0.001
Something else	1.88	(1.78, 2.01)	<0.001	None of these	1.14	(1.07, 1.22)	<0.001
Interactions (ref = no, straight)				Interactions (ref = no, male)			
Gay or lesbian	0.80	(0.72, 0.9)	<0.001	Female	0.99	(0.96, 1.02)	0.45
Bisexual	0.81	(0.77, 0.87)	<0.001	Transgender	0.61	(0.5, 0.76)	<0.001
Something else	0.79	(0.71, 0.88)	<0.001	None of these	0.79	(0.7, 0.9)	<0.001
**Effect measure modifications by pandemic-induced housing instability**							
**Variables**	**OR**	(**95% CI**)	***p***	**Variables**	**OR**	(**95% CI**)	***p***
Housing instability (ref = no)				Housing instability (ref = no)			
Yes	0.88	(0.86, 0.9)	<0.001	Yes	0.92	(0.89, 0.95)	<0.001
Sexual orientation (ref = straight)				Gender identity (ref = male)			
Gay or lesbian	3.31	(3.11, 3.54)	<0.001	Female	1.12	(1.11, 1.14)	<0.001
Bisexual	2.34	(2.25, 2.44)	<0.001	Transgender	1.96	(1.73, 2.24)	<0.001
Something else	1.82	(1.71, 1.94)	<0.001	None of these	1.10	(1.04, 1.17)	0.00
Interactions (ref = no, straight)				Interactions (ref = no, male)			
Gay or lesbian	0.66	(0.59, 0.74)	<0.001	Female	0.90	(0.88, 0.94)	<0.001
Bisexual	0.66	(0.61, 0.73)	<0.001	Transgender	0.54	(0.38, 0.78)	0.00
Something else	0.85	(0.72, 1.03)	0.08	None of these	0.86	(0.72, 1.04)	0.11
**Effect measure modifications by pandemic-induced food insufficiency**							
**Variables**	**OR**	(**95% CI**)	***p***	**Variables**	**OR**	(**95% CI**)	***p***
Food insufficiency (ref = no)				Food insufficiency (ref = no)			
Yes	0.73	(0.72, 0.75)	<0.001	Yes	0.70	(0.69, 0.72)	<0.001
Sexual orientation (ref = straight)				Gender identity (ref = male)			
Gay or Lesbian	3.63	(3.32, 3.99)	<0.001	Female	1.08	(1.06, 1.11)	<0.001
Bisexual	2.50	(2.37, 2.65)	<0.001	Transgender	1.60	(1.33, 1.95)	<0.001
Something else	1.74	(1.61, 1.9)	<0.001	None of these	0.91	(0.83, 1.01)	0.05
Interactions (ref = no, straight)				Interactions (ref = no, male)			
Gay or Lesbian	0.78	(0.72, 0.86)	<0.001	Female	1.07	(1.05, 1.1)	<0.001
Bisexual	0.83	(0.79, 0.88)	<0.001	Transgender	1.22	(0.98, 1.54)	0.08
Something else	1.04	(0.94, 1.15)	0.49	None of these	1.35	(1.2, 1.53)	<0.001
**Effect measure modifications by pandemic-induced household expense difficulty**							
**Variables**	**OR**	(**95% CI**)	***p***	**Variables**	**OR**	(**95% CI**)	***p***
HH Expense Difficulty (ref = no)				HH expense difficulty (ref = no)			
Yes	0.73	(0.72, 0.75)	<0.001	Yes	0.69	(0.68, 0.72)	<0.001
Sexual Orientation (ref = straight)				Gender identity (ref = male)			
Gay or Lesbian	3.76	(3.37, 4.22)	<0.001	Female	1.05	(1.03, 1.07)	<0.001
Bisexual	2.43	(2.27, 2.61)	<0.001	Transgender	1.00	(0.77, 1.31)	0.99
Something else	1.40	(1.27, 1.56)	<0.001	None of these	0.72	(0.65, 0.82)	<0.001
Interactions (ref = no, straight)				Interactions (ref = no, male)			
Gay or Lesbian	0.80	(0.72, 0.89)	<0.001	Female	1.09	(1.07, 1.12)	<0.001
Bisexual	0.90	(0.85, 0.96)	0.00	Transgender	2.10	(1.62, 2.73)	<0.001
Something else	1.35	(1.24, 1.47)	<0.001	None of these	1.69	(1.5, 1.92)	<0.001

Note: OR = odds ratio. CI = confidence interval. Ref = reference group. HH = household. All models were adjusted for employment income loss, housing instability, food insufficiency, household expense difficulty, sexual orientation, gender identity, race/ethnicity, age group, marital status, children, household size, education, household income, work status, health insurance, anxiety, depression, place of residence, and survey week. Standard errors were clustered at the state level. This table shows a summary of eight different model estimates and full results are shown in Supplemental Tables S3 and S4.

## Data Availability

Data are contained within the article and Supplementary Materials.

## Supplementary Materials

The following supporting information can be downloaded at https://www.mdpi.com/article/10.3390/vaccines12111277/s1: Table S1: U.S. Census Bureau’s Household Pulse Survey microdata sample size by survey week; Table S2: Data source, survey questionnaire, and survey answer options for variables in the model; Table S3: Full model results on the effect measure modification by sexual orientation on the association between pandemic-induced socioeconomic hardship and COVID-19 vaccine uptake; Table S4: Full model results on the effect measure modification by gender identity between pandemic-induced socioeconomic hardship and COVID-19 vaccine uptake.
