# Supplementary material for: Heterogeneous Associations Between Pandemic-Induced Socioeconomic Hardships and COVID-19 Vaccine Uptake by Sexual Orientation and Gender Identity: A Nationally Representative Analysis in the United States
Source: Vaccines (Basel). 2024 Nov 13;12(11):1277. doi: 10.3390/vaccines12111277 (PMC11598954; doi:10.3390/vaccines12111277)
Supplement: Supplementary file 1 [file vaccines-12-01277-s001.zip › vaccines-3242529-supplementary.pdf]

Supplemental Materials

Supplemental tables of the main article are presented in this material.

Supplemental Table S1. U.S. Census Bureau's Household Pulse Survey Microdata Sample Size by Survey Week, 21 July 2021–24 June 2024.

Survey Phase	Survey Week	Survey Dates	Sample Size		
			Count	Cumulative	%
3.2	34	July 21 – August 2, 2021	64,562	64,562	2.6
	35	August 4 – August 16	68,799	133,361	2.8
	36	August 18 – August 30	69,114	202,475	2.8
	37	September 1 - September 13	63,536	266,011	2.6
	38	September 15 – September 27	59,833	325,844	2.4
	39	September 29 - October 11	57,064	382,908	2.3
3.3	40	December 1 – December 13	60,826	443,734	2.5
	41	December 29, 2021 – January 10, 2022	74,995	518,729	3.1
	42	January 26 – February 7, 2022	75,482	594,211	3.1
3.4	43	March 2 – March 14	84,158	678,369	3.4
	44	March 30 – April 11	63,769	742,138	2.6
	45	April 27 – May 9	61,767	803,905	2.5
3.5	46	June 1 - June 13	62,826	866,731	2.6
	47	June 29 - July 11	58,304	925,035	2.4
	48	July 27 - August 8	46,801	971,836	1.9
3.6	49	September 14 - September 28	50,937	1,022,773	2.1
	50	October 5 - October 17	42,040	1,064,813	1.7
	51	November 2 - November 14	61,453	1,126,266	2.5
3.7	52	December 9 - December 19, 2022	70,685	1,196,951	2.9
	53	January 4 - January 16, 2023	68,504	1,265,455	2.8
	54	February 1 - February 13	75,709	1,341,164	3.1
3.8	55	March 1 - March 13	72,738	1,413,902	3.0
	56	March 29 - April 10	61,927	1,475,829	2.5
	57	April 26 - May 8	59,290	1,535,119	2.4
3.9	58	June 7 – June 19	64,792	1,599,911	2.6
	59	June 28 – July 10	63,802	1,663,713	2.6
	60	July 26 – August 7	68,830	1,732,543	2.8
3.10	61	August 23 – September 4	68,454	1,800,997	2.8
	62	September 20 - October 2	72,839	1,873,836	3.0
	63	October 18 - October 30, 2023	79,371	1,953,207	3.2
4.0	Cycle 1	January 9 - February 5, 2024	68,544	2,021,751	2.8
	Cycle 2	February 6 - March 4	71,152	2,092,903	2.9
	Cycle 3	March 5 - April 1	71,798	2,164,701	2.9
4.1	Cycle 4	April 2 - April 29	73,352	2,238,053	3.0
	Cycle 5	April 30 - May 27	72,162	2,310,215	2.9
	Cycle 6	May 28 - June 24	72,125	2,382,340	2.9
	Cycle 7	June 25 - July 22, 2024	70,429	2,452,769	2.9

Note: This table shows the unweighted number of survey samples. If weighted by person-weight (*pweight* in HPS microdata), each survey week represents the adult (ages 18+) population of 250,265,449 in 2021, 252,481,011 in 2022, 254,966,752 in 2023, and 256,311,560 in 2024. If weighted by household-weight (*hweight* in HPS microdata), each week represents the total households of 122,798,192 in 2021, 124,345,410 in 2022, 127,544,730 in 2023, and 129,870,928 in 2024. Source: U.S. Census Bureau's Household Pulse Survey (HPS) Public Use File (PUF).

Supplemental Table S2. Data Source, Survey Questionnaire, and Survey Answer Options for Variables in the Model.

Variable	Survey Questionnaire	Survey Answer Options
**Dependent Variables**		
COVID-19 vaccine uptake	Have you received a COVID-19 vaccine?	1) Yes<br>2) No
**Independent Variables**		
Sexual orientation and gender identity		
Sexual orientation	Which of the following best represents how you think of yourself?	1) Gay or lesbian<br>2) Straight, that is not gay or lesbian<br>3) Bisexual<br>4) Something else, please specify_____________<br>5) I don't know<br>-99) Question seen but category not selected<br>-88) Missing / Did not report
Gender identity	Do you currently describe yourself as male, female, or transgender?	1) Male<br>2) Female<br>3) Transgender<br>4) None of these<br>-99) Question seen but category not selected<br>-88) Missing / Did not report
COVID-19 pandemic-induced hardships		
Employment income loss	Have you, or has anyone in your household experienced a loss of employment income in the last 4 weeks?	1) Yes<br>2) No
Housing instability	Is this household currently caught up on mortgage payments?	1) Yes<br>2) No
Food insufficiency	In the last 7 days, which of these statements best describes the food eaten in your household?	1) Enough of the kinds of food (I/we) wanted to eat<br>2) Enough, but not always the kinds of food (I/we) wanted to eat<br>3) Sometimes not enough to eat<br>4) Often not enough to eat
Household expense difficulty	In the last 7 days, how difficult has it been for your household to pay for usual household expenses, including but not limited to food, rent or mortgage, car payments, medical expenses, student loans, and so on?	1) Not at all<br>2) Several days<br>3) More than half the days<br>4) Nearly every day \
Individual and Household-level Covariates		
Race/ethnicity	Are you of Hispanic, Latino, or Spanish origin? - Selected Choice	1) No, not of Hispanic, Latino, or Spanish origin<br>2) Yes, of Hispanic, Latino, or Spanish origin

Variable	Survey Questionnaire	Survey Answer Options
	What is your race? Please select all that apply. - Selected Choice	1) White, Alone<br>2) Black, Alone<br>3) Asian, Alone<br>4) Any other race alone, or race in combination
Age	What year were you born? Please enter a number.	1932-2002
Marital status	What is your marital status? Select only one answer.	1) Now married<br>2) Widowed<br>3) Divorced<br>4) Separated<br>5) Never married
Children in household	How many people under 18 years-old currently live in your household? Please enter a number.	(0-40) number of people under 18 (whole number)
Household size	How many total people – adults and children – currently live in your household, including yourself? Please enter a number.	(1-40) number of people (whole number)
Education	What is the highest degree or level of school you have completed? Select only one answer.	1) Less than high school<br>2) Some high school<br>3) High school graduate or equivalent (for example GED)<br>4) Some college, but degree not received or is in progress<br>5) Associate's degree (for example AA, AS)<br>6) Bachelor's degree (for example BA, BS, AB)<br>7) Graduate degree (for example master's, professional, doctorate)
Household income	In 2019 what was your total household income before taxes? Select only one answer.	1) Less than $25,000<br>2) $25,000 - $34,999<br>3) $35,000 - $49,999<br>4) $50,000 - $74,999<br>5) $75,000 - $99,999<br>6) $100,000 - $149,999<br>7) $150,000 - $199,999<br>8) $200,000 and above
Work status	Are you employed by government, by a private company, a nonprofit organization or were you self-	1) Government<br>2) Private company<br>3) Non-profit organization including tax exempt and

Variable	Survey Questionnaire	Survey Answer Options
	employed or working in a family business? Select only one answer.	charitable organizations<br>4) Self-employed<br>5) Working in a family business
Health insurance	Are you currently covered by any of the following types of health<br>insurance or health coverage plans? Mark Yes or No for each. - Insurance through a current or former employer or union (through yourself or another family member)	1) Insurance through a current or former employer or union (through yourself or another family member)<br>2) Insurance purchased directly from an insurance company, including marketplace coverage (through yourself or another family member)<br>3) Medicare, for people 65 and older, or people with certain disabilities<br>4) Medicaid, Medical Assistance, or any kind of government-assistance plan for those with low incomes or a disability<br>5) TRICARE or other military health care<br>6) VA (including those who have ever used or enrolled for VA health care)<br>7) Indian Health Service<br>8) Other
Anxiety	Over the last 7 days, how often have you been bothered by the following problems ... Feeling nervous, anxious, or on edge? Would you say not at all, several days, more than half the days, or nearly every day? Select only one answer.	1) Not at all<br>2) Several days<br>3) More than half the days<br>4) Nearly every day\
	Over the last 7 days, how often have you been bothered by the following problems ... Not being able to stop or control worrying? Would you say not at all, several days, more than half the days, or nearly every day? Select only one answer.	1) Not at all<br>2) Several days<br>3) More than half the days<br>4) Nearly every day\
Depression	Over the last 7 days, how often have you been bothered by ... having little interest or pleasure in doing things? Would you say not at all, several days, more than half the days, or nearly every day? Select only one answer.	1) Not at all<br>2) Several days<br>3) More than half the days<br>4) Nearly every day\
	Over the last 7 days, how often have you been bothered by ... feeling down, depressed, or hopeless? Would you say not at all, several days, more than half the days, or nearly every day? Select only one answer.	1) Not at all<br>2) Several days<br>3) More than half the days<br>4) Nearly every day\
Spatial and Temporal Fixed-Effects		
Metropolitan area	Data based on the HPS record	-
Survey week	Data based on the HPS record	-

Notes: HPS = Household Pulse Survey.

Supplemental Table S3. Full model results on the effect measure modification by sexual orientation on the association between pandemic-induced socioeconomic hardship and COVID-19 vaccine uptake, 21 July 2021—24 June 2024 (*N* = 1,767,966).

Variables	Employment income loss × Sexual orientation			Housing instability × Sexual orientation			Food insufficiency × Sexual orientation			HH expense difficulty × Sexual orientation		
	OR		(95% CI)	OR		(95% CI)	OR		(95% CI)	OR		(95% CI)
**Interactions**												
Gay or lesbian	0.80	***	(0.72, 0.9)	0.66	***	(0.59, 0.74)	0.78	***	(0.72, 0.86)	0.80	***	(0.72, 0.89)
Bisexual	0.81	***	(0.77, 0.87)	0.66	***	(0.61, 0.73)	0.83	***	(0.79, 0.88)	0.90	**	(0.85, 0.96)
Something else	0.79	***	(0.71, 0.88)	0.85	+	(0.72, 1.03)	1.04		(0.94, 1.15)	1.35	***	(1.24, 1.47)
**Employment Income loss (ref = no)**												
Yes	0.95	***	(0.93, 0.97)	0.93	***	(0.92, 0.95)	0.93	***	(0.92, 0.95)	0.93	***	(0.91, 0.95)
**Housing instability (ref = no)**												
Yes	0.85	***	(0.84, 0.87)	0.88	***	(0.86, 0.9)	0.85	***	(0.84, 0.87)	0.85	***	(0.84, 0.87)
**Food insufficiency (ref = no)**												
Yes	0.73	***	(0.72, 0.75)	0.73	***	(0.72, 0.75)	0.73	***	(0.72, 0.75)	0.73	***	(0.72, 0.75)
**Household Expense difficulty (ref = no)**												
Yes	0.73	***	(0.72, 0.75)	0.73	***	(0.72, 0.75)	0.73	***	(0.72, 0.75)	0.73	***	(0.72, 0.75)
**Sexual orientation (ref = straight)**												
Gay or lesbian	3.30	***	(3.07, 3.56)	3.31	***	(3.11, 3.54)	3.63	***	(3.32, 3.99)	3.76	***	(3.37, 4.22)
Bisexual	2.32	***	(2.23, 2.42)	2.34	***	(2.25, 2.44)	2.50	***	(2.37, 2.65)	2.43	***	(2.27, 2.61)
Something else	1.88	***	(1.78, 2.01)	1.82	***	(1.71, 1.94)	1.74	***	(1.61, 1.9)	1.40	***	(1.27, 1.56)
**Gender identity (ref = male)**												
Female	1.11	***	(1.1, 1.13)	1.11	***	(1.1, 1.13)	1.11	***	(1.1, 1.13)	1.11	***	(1.1, 1.13)
Transgender	1.84	***	(1.61, 2.1)	1.82	***	(1.6, 2.09)	1.84	***	(1.62, 2.11)	1.82	***	(1.6, 2.08)
None of these	1.09	**	(1.03, 1.16)	1.08	*	(1.02, 1.15)	1.09	**	(1.03, 1.16)	1.08	*	(1.02, 1.15)
**Race/ethnicity (ref = White)**												
Non-Hispanic Black	1.56	***	(1.39, 1.75)	1.55	***	(1.39, 1.75)	1.56	***	(1.39, 1.75)	1.56	***	(1.39, 1.75)
Non-Hispanic A&PI	3.65	***	(3.29, 4.05)	3.64	***	(3.28, 4.05)	3.65	***	(3.29, 4.05)	3.65	***	(3.29, 4.05)
Non-Hispanic others	0.98		(0.91, 1.06)	0.98		(0.91, 1.06)	0.98		(0.91, 1.06)	0.98		(0.91, 1.06)
Hispanic	1.62	***	(1.48, 1.78)	1.62	***	(1.48, 1.78)	1.62	***	(1.48, 1.78)	1.62	***	(1.48, 1.78)
**Age (ref = 18-24)**												
25-34	0.85	***	(0.81, 0.9)	0.85	***	(0.82, 0.9)	0.85	***	(0.81, 0.9)	0.85	***	(0.81, 0.9)
35-44	1.05		(0.99, 1.12)	1.05		(0.99, 1.12)	1.05		(0.99, 1.12)	1.05		(0.99, 1.12)
45-54	1.21	***	(1.15, 1.28)	1.21	***	(1.15, 1.28)	1.21	***	(1.15, 1.28)	1.21	***	(1.15, 1.28)
55-64	1.67	***	(1.58, 1.78)	1.68	***	(1.58, 1.79)	1.67	***	(1.57, 1.78)	1.67	***	(1.58, 1.78)
65-74	3.19	***	(2.96, 3.45)	3.20	***	(2.97, 3.46)	3.18	***	(2.95, 3.44)	3.19	***	(2.96, 3.45)
75+	4.49	***	(4.09, 4.93)	4.50	***	(4.1, 4.94)	4.48	***	(4.08, 4.92)	4.48	***	(4.09, 4.93)
**Marital status (ref = unmarried)**												
Married	1.01		(0.99, 1.04)	1.01		(0.99, 1.04)	1.01		(0.99, 1.04)	1.01		(0.99, 1.04)

Variables	Employment income loss × Sexual orientation			Housing instability × Sexual orientation			Food insufficiency × Sexual orientation			HH expense difficulty × Sexual orientation		
	OR		(95% CI)	OR		(95% CI)	OR		(95% CI)	OR		(95% CI)
**Children in household (ref = no child)**												
One or more children	0.81	***	(0.8, 0.83)	0.81	***	(0.8, 0.83)	0.81	***	(0.8, 0.83)	0.81	***	(0.8, 0.83)
**Household size (ref = single person)**												
2-person	0.96	***	(0.95, 0.98)	0.96	***	(0.95, 0.98)	0.96	***	(0.95, 0.98)	0.96	***	(0.95, 0.98)
3-person	0.87	***	(0.86, 0.9)	0.87	***	(0.85, 0.9)	0.87	***	(0.85, 0.9)	0.87	***	(0.86, 0.9)
4-person	0.81	***	(0.79, 0.83)	0.81	***	(0.79, 0.83)	0.81	***	(0.79, 0.83)	0.81	***	(0.79, 0.83)
5-person	0.70	***	(0.68, 0.73)	0.70	***	(0.68, 0.73)	0.70	***	(0.68, 0.73)	0.70	***	(0.68, 0.73)
6 or more persons	0.57	***	(0.55, 0.61)	0.57	***	(0.55, 0.61)	0.57	***	(0.55, 0.61)	0.57	***	(0.55, 0.61)
**Education (ref = less than High School)**												
High school	1.08	*	(1.02, 1.16)	1.08	*	(1.02, 1.16)	1.08	*	(1.02, 1.16)	1.08	*	(1.02, 1.16)
Some college & AA	1.50	***	(1.4, 1.62)	1.50	***	(1.4, 1.62)	1.50	***	(1.4, 1.62)	1.50	***	(1.4, 1.62)
BA+	3.20	***	(2.96, 3.46)	3.20	***	(2.96, 3.46)	3.20	***	(2.96, 3.47)	3.20	***	(2.96, 3.47)
**Household income (ref = less than $25,000)**												
$25,000–49,999	1.20	***	(1.18, 1.23)	1.20	***	(1.18, 1.23)	1.20	***	(1.18, 1.23)	1.20	***	(1.18, 1.23)
$50,000–74,999	1.25	***	(1.22, 1.28)	1.25	***	(1.22, 1.28)	1.25	***	(1.22, 1.28)	1.25	***	(1.22, 1.28)
$75,000–99,999	1.32	***	(1.28, 1.37)	1.32	***	(1.28, 1.37)	1.32	***	(1.28, 1.37)	1.32	***	(1.28, 1.37)
$100,000–$149,999	1.52	***	(1.47, 1.58)	1.52	***	(1.47, 1.58)	1.52	***	(1.47, 1.58)	1.52	***	(1.47, 1.58)
$150,000 and above	2.11	***	(1.99, 2.24)	2.11	***	(1.99, 2.24)	2.11	***	(1.99, 2.24)	2.11	***	(1.99, 2.24)
**Work status (ref = no work)**												
Government	1.33	***	(1.28, 1.39)	1.33	***	(1.28, 1.39)	1.33	***	(1.28, 1.39)	1.33	***	(1.28, 1.39)
Private company	1.00		(0.98, 1.03)	1.00		(0.98, 1.03)	1.00		(0.98, 1.03)	1.00		(0.98, 1.03)
Non-profit	1.79	***	(1.71, 1.89)	1.79	***	(1.71, 1.89)	1.79	***	(1.71, 1.89)	1.79	***	(1.71, 1.89)
Others	0.66	***	(0.64, 0.69)	0.66	***	(0.64, 0.69)	0.66	***	(0.64, 0.69)	0.66	***	(0.64, 0.69)
**Health insurance (ref = public)**												
Private	1.35	***	(1.33, 1.38)	1.35	***	(1.33, 1.38)	1.35	***	(1.32, 1.38)	1.35	***	(1.33, 1.38)
Both	1.24	***	(1.23, 1.27)	1.24	***	(1.23, 1.27)	1.24	***	(1.23, 1.27)	1.24	***	(1.23, 1.27)
None	0.75	***	(0.73, 0.78)	0.75	***	(0.73, 0.78)	0.75	***	(0.73, 0.78)	0.75	***	(0.73, 0.78)
**Anxiety (ref = no)**												
Yes	1.23	***	(1.22, 1.26)	1.23	***	(1.22, 1.26)	1.23	***	(1.22, 1.26)	1.23	***	(1.22, 1.26)
**Depression (ref = no)**												
Yes	1.05	***	(1.03, 1.07)	1.05	***	(1.03, 1.07)	1.05	***	(1.03, 1.07)	1.05	***	(1.03, 1.07)
**Place of residence (ref = non-metro)**												
Metropolitan Area	1.32	***	(1.17, 1.5)	1.32	***	(1.17, 1.5)	1.32	***	(1.17, 1.5)	1.32	***	(1.17, 1.5)
**Survey week (ref = July 21–August 2, 2021)**												
August 4 – August 16	1.01		(0.98, 1.05)	1.01		(0.98, 1.05)	1.01		(0.98, 1.05)	1.01		(0.98, 1.05)
August 18 – August 30	1.09	**	(1.04, 1.15)	1.09	**	(1.04, 1.15)	1.09	**	(1.04, 1.15)	1.09	**	(1.04, 1.15)

Variables	Employment income loss × Sexual orientation			Housing instability × Sexual orientation			Food insufficiency × Sexual orientation			HH expense difficulty × Sexual orientation		
	OR		(95% CI)	OR		(95% CI)	OR		(95% CI)	OR		(95% CI)
September 1 - September 13	1.14	***	(1.09, 1.2)	1.14	***	(1.09, 1.2)	1.14	***	(1.09, 1.2)	1.14	***	(1.09, 1.2)
September 15 – September 27	1.12	***	(1.07, 1.19)	1.12	***	(1.07, 1.19)	1.12	***	(1.07, 1.19)	1.12	***	(1.07, 1.19)
September 29 - October 11	1.16	***	(1.1, 1.24)	1.16	***	(1.1, 1.24)	1.16	***	(1.1, 1.24)	1.16	***	(1.1, 1.24)
December 1 – December 13	1.30	***	(1.24, 1.39)	1.30	***	(1.24, 1.39)	1.30	***	(1.24, 1.39)	1.30	***	(1.24, 1.39)
December 29, 2021 – January 10, 2022	1.37	***	(1.3, 1.46)	1.37	***	(1.3, 1.45)	1.37	***	(1.3, 1.46)	1.37	***	(1.3, 1.46)
January 26 – February 7, 2022	1.36	***	(1.28, 1.45)	1.36	***	(1.28, 1.45)	1.36	***	(1.28, 1.45)	1.36	***	(1.28, 1.45)
March 2 – March 14	1.41	***	(1.34, 1.5)	1.41	***	(1.34, 1.5)	1.41	***	(1.34, 1.5)	1.41	***	(1.34, 1.5)
March 30 – April 11	1.28	***	(1.22, 1.36)	1.28	***	(1.22, 1.36)	1.28	***	(1.22, 1.36)	1.28	***	(1.22, 1.36)
April 27 – May 9	1.29	***	(1.23, 1.36)	1.29	***	(1.23, 1.36)	1.29	***	(1.23, 1.36)	1.29	***	(1.23, 1.36)
June 1 - June 13	1.28	***	(1.2, 1.36)	1.28	***	(1.2, 1.36)	1.27	***	(1.2, 1.36)	1.27	***	(1.2, 1.36)
June 29 - July 11	1.29	***	(1.24, 1.36)	1.29	***	(1.24, 1.36)	1.29	***	(1.24, 1.36)	1.29	***	(1.24, 1.36)
July 27 - August 8	1.17	***	(1.11, 1.25)	1.17	***	(1.11, 1.25)	1.17	***	(1.11, 1.25)	1.17	***	(1.11, 1.25)
September 14 - September 28	1.10	***	(1.05, 1.17)	1.10	**	(1.05, 1.17)	1.10	**	(1.05, 1.17)	1.10	***	(1.05, 1.17)
October 5 - October 17	1.07	*	(1.01, 1.14)	1.07	*	(1.01, 1.14)	1.07	*	(1.01, 1.14)	1.07	*	(1.01, 1.14)
November 2 - November 14	1.08	*	(1.02, 1.15)	1.08	*	(1.02, 1.15)	1.08	*	(1.02, 1.15)	1.08	*	(1.02, 1.15)
December 9 - December 19, 2022	1.13	***	(1.08, 1.2)	1.13	***	(1.08, 1.2)	1.13	***	(1.08, 1.2)	1.13	***	(1.08, 1.2)
January 4 - January 16, 2023	1.14	***	(1.09, 1.2)	1.14	***	(1.09, 1.2)	1.14	***	(1.09, 1.2)	1.14	***	(1.09, 1.2)
February 1 - February 13	1.13	***	(1.08, 1.2)	1.13	***	(1.08, 1.2)	1.13	***	(1.08, 1.2)	1.13	***	(1.08, 1.2)
March 1 - March 13	1.11	**	(1.05, 1.19)	1.11	**	(1.05, 1.19)	1.11	**	(1.05, 1.19)	1.11	**	(1.05, 1.19)
March 29 - April 10	1.11	***	(1.06, 1.18)	1.11	***	(1.06, 1.18)	1.11	***	(1.06, 1.18)	1.11	***	(1.06, 1.18)
April 26 - May 8	1.11	***	(1.05, 1.17)	1.11	***	(1.05, 1.17)	1.11	***	(1.05, 1.17)	1.11	***	(1.05, 1.17)
June 7 – June 19	1.12	***	(1.07, 1.19)	1.12	***	(1.07, 1.19)	1.12	***	(1.07, 1.19)	1.12	***	(1.07, 1.19)
June 28 – July 10	1.10	***	(1.05, 1.16)	1.10	***	(1.05, 1.16)	1.10	***	(1.05, 1.16)	1.10	***	(1.05, 1.16)
July 26 – August 7	1.09	**	(1.04, 1.15)	1.09	**	(1.04, 1.15)	1.09	**	(1.04, 1.15)	1.09	**	(1.04, 1.15)
August 23 – September 4	1.08	*	(1.02, 1.16)	1.08	*	(1.02, 1.16)	1.08	*	(1.02, 1.16)	1.08	*	(1.02, 1.16)
September 20 - October 2	1.10	**	(1.04, 1.18)	1.10	**	(1.04, 1.17)	1.10	**	(1.04, 1.18)	1.10	**	(1.04, 1.17)
October 18 - October 30, 2023	1.07	*	(1.02, 1.13)	1.07	*	(1.02, 1.13)	1.07	*	(1.02, 1.13)	1.07	*	(1.02, 1.13)
January 9 - February 5, 2024	0.93	*	(0.89, 1)	0.93	*	(0.89, 1)	0.93	*	(0.89, 1)	0.93	*	(0.89, 1)
February 6 - March 4	0.90	***	(0.85, 0.96)	0.90	***	(0.85, 0.96)	0.90	***	(0.85, 0.96)	0.90	***	(0.85, 0.96)
March 5 - April 1	0.91	***	(0.86, 0.96)	0.91	***	(0.86, 0.96)	0.91	**	(0.86, 0.96)	0.91	**	(0.86, 0.96)
April 2 - April 29	0.94	*	(0.89, 1)	0.94	*	(0.89, 1)	0.94	*	(0.89, 1)	0.94	*	(0.89, 1)
April 30 - May 27	0.92	**	(0.87, 0.98)	0.92	**	(0.87, 0.98)	0.92	**	(0.87, 0.98)	0.92	**	(0.87, 0.98)
May 28 - June 24	0.93	*	(0.89, 0.99)	0.93	*	(0.89, 0.99)	0.93	*	(0.89, 0.99)	0.93	*	(0.89, 0.99)
June 25 - July 22, 2024	0.93	**	(0.89, 0.98)	0.93	**	(0.89, 0.98)	0.93	**	(0.89, 0.98)	0.93	**	(0.89, 0.98)
**Constant**	1.85	***	(1.55, 2.21)	1.84	***	(1.55, 2.21)	1.84	***	(1.55, 2.21)	1.85	***	(1.55, 2.21)

Note: OR = Odds ratio. CI = Confidence interval. Ref = Reference group. A&PI = Asian and Pacific Islander. AA = Associate in Arts. BA+ = Bachelor's degree or higher. HH = Household. MSA = Metropolitan statistical area. Standard errors were clustered at the state level.

Supplemental Table S4. Full model results on the effect measure modification by gender identity between pandemic-induced socioeconomic hardship and COVID-19 vaccine uptake, 21 July 2021—24 June 2024 (*N* = 1,767,966).

Variables	Employment income loss × Gender identity			Housing instability × Gender identity			Food insufficiency × Gender identity			HH expense difficulty × Gender identity		
	OR		(95% CI)	OR		(95% CI)	OR		(95% CI)	OR		(95% CI)
**Interactions**												
Female	0.99		(0.96, 1.02)	0.90	***	(0.88, 0.94)	1.07	***	(1.05, 1.1)	1.09	***	(1.07, 1.12)
Transgender	0.61	***	(0.5, 0.76)	0.54	**	(0.38, 0.78)	1.22	+	(0.98, 1.54)	2.10	***	(1.62, 2.73)
None of these	0.79	***	(0.7, 0.9)	0.86		(0.72, 1.04)	1.35	***	(1.2, 1.53)	1.69	***	(1.5, 1.92)
**Employment Income loss (ref = no)**												
Yes	0.94	***	(0.92, 0.97)	0.93	***	(0.91, 0.95)	0.93	***	(0.92, 0.95)	0.93	***	(0.92, 0.95)
**Housing instability (ref = no)**												
Yes	0.85	***	(0.84, 0.87)	0.92	***	(0.89, 0.95)	0.85	***	(0.84, 0.87)	0.85	***	(0.83, 0.87)
**Food insufficiency (ref = no)**												
Yes	0.73	***	(0.72, 0.75)	0.73	***	(0.72, 0.75)	0.70	***	(0.69, 0.72)	0.73	***	(0.72, 0.75)
**Household Expense difficulty (ref = no)**												
Yes	0.73	***	(0.72, 0.75)	0.73	***	(0.72, 0.75)	0.73	***	(0.72, 0.75)	0.69	***	(0.68, 0.72)
**Sexual orientation (ref = straight)**												
Gay or lesbian	1.11	***	(1.1, 1.13)	1.12	***	(1.11, 1.14)	1.08	***	(1.06, 1.11)	1.05	***	(1.03, 1.07)
Bisexual	2.08	***	(1.8, 2.4)	1.96	***	(1.73, 2.24)	1.60	***	(1.33, 1.95)	1.00		(0.77, 1.31)
Something else	1.14	***	(1.07, 1.22)	1.10	**	(1.04, 1.17)	0.91	+	(0.83, 1.01)	0.72	***	(0.65, 0.82)
**Gender identity (ref = male)**												
Female	3.17	***	(2.97, 3.39)	3.16	***	(2.97, 3.39)	3.17	***	(2.97, 3.39)	3.17	***	(2.97, 3.39)
Transgender	2.23	***	(2.15, 2.32)	2.23	***	(2.15, 2.32)	2.22	***	(2.14, 2.31)	2.22	***	(2.15, 2.31)
None of these	1.79	***	(1.68, 1.92)	1.79	***	(1.68, 1.91)	1.78	***	(1.67, 1.91)	1.78	***	(1.67, 1.91)
**Race/ethnicity (ref = White)**												
Non-Hispanic Black	1.56	***	(1.39, 1.75)	1.56	***	(1.39, 1.75)	1.55	***	(1.39, 1.75)	1.55	***	(1.39, 1.75)
Non-Hispanic A&PI	3.65	***	(3.29, 4.05)	3.64	***	(3.28, 4.05)	3.65	***	(3.29, 4.06)	3.65	***	(3.29, 4.06)
Non-Hispanic others	0.98		(0.91, 1.06)	0.98		(0.91, 1.06)	0.98		(0.91, 1.06)	0.98		(0.91, 1.06)
Hispanic	1.62	***	(1.48, 1.78)	1.62	***	(1.48, 1.78)	1.62	***	(1.48, 1.78)	1.62	***	(1.48, 1.78)
**Age (ref = 18-24)**												
25-34	0.85	***	(0.81, 0.9)	0.85	***	(0.82, 0.9)	0.85	***	(0.81, 0.9)	0.85	***	(0.81, 0.9)
35-44	1.05		(0.99, 1.12)	1.05		(0.99, 1.12)	1.05		(0.99, 1.12)	1.05		(0.99, 1.12)
45-54	1.21	***	(1.15, 1.28)	1.21	***	(1.15, 1.28)	1.21	***	(1.15, 1.28)	1.21	***	(1.15, 1.28)
55-64	1.67	***	(1.58, 1.78)	1.67	***	(1.58, 1.78)	1.67	***	(1.58, 1.78)	1.67	***	(1.58, 1.78)
65-74	3.19	***	(2.96, 3.45)	3.19	***	(2.96, 3.45)	3.19	***	(2.96, 3.45)	3.19	***	(2.96, 3.45)
75+	4.49	***	(4.09, 4.93)	4.48	***	(4.09, 4.93)	4.48	***	(4.08, 4.92)	4.48	***	(4.08, 4.92)
**Marital status (ref = unmarried)**												
Married	1.01		(0.99, 1.04)	1.01		(0.99, 1.04)	1.01		(0.99, 1.04)	1.01		(0.99, 1.04)

Variables	Employment income loss × Gender identity			Housing instability × Gender identity			Food insufficiency × Gender identity			HH expense difficulty × Gender identity		
	OR		(95% CI)	OR		(95% CI)	OR		(95% CI)	OR		(95% CI)
**Children in household (ref = no child)**												
One or more children	0.81	***	(0.8, 0.83)	0.81	***	(0.8, 0.83)	0.81	***	(0.8, 0.82)	0.81	***	(0.8, 0.82)
**Household size (ref = single person)**												
2-person	0.96	***	(0.95, 0.98)	0.96	***	(0.95, 0.99)	0.96	***	(0.94, 0.98)	0.96	***	(0.94, 0.98)
3-person	0.87	***	(0.86, 0.9)	0.87	***	(0.86, 0.9)	0.87	***	(0.85, 0.9)	0.87	***	(0.85, 0.9)
4-person	0.81	***	(0.79, 0.83)	0.81	***	(0.79, 0.83)	0.81	***	(0.79, 0.83)	0.81	***	(0.79, 0.83)
5-person	0.70	***	(0.68, 0.73)	0.70	***	(0.68, 0.74)	0.70	***	(0.68, 0.73)	0.70	***	(0.68, 0.73)
6 or more persons	0.57	***	(0.55, 0.61)	0.57	***	(0.55, 0.61)	0.57	***	(0.55, 0.6)	0.57	***	(0.55, 0.61)
**Education (ref = less than High School)**												
High school	1.08	*	(1.02, 1.16)	1.08	*	(1.02, 1.16)	1.08	*	(1.02, 1.16)	1.08	*	(1.02, 1.16)
Some college & AA	1.50	***	(1.4, 1.62)	1.50	***	(1.4, 1.62)	1.50	***	(1.4, 1.62)	1.50	***	(1.4, 1.62)
BA+	3.20	***	(2.96, 3.47)	3.20	***	(2.96, 3.47)	3.20	***	(2.96, 3.47)	3.20	***	(2.96, 3.46)
**Household income (ref = less than $25,000)**												
$25,000–49,999	1.20	***	(1.18, 1.23)	1.20	***	(1.18, 1.23)	1.20	***	(1.18, 1.23)	1.20	***	(1.18, 1.23)
$50,000–74,999	1.25	***	(1.22, 1.28)	1.25	***	(1.22, 1.28)	1.25	***	(1.23, 1.29)	1.25	***	(1.23, 1.29)
$75,000–99,999	1.32	***	(1.28, 1.37)	1.32	***	(1.28, 1.37)	1.32	***	(1.28, 1.38)	1.33	***	(1.29, 1.38)
$100,000–$149,999	1.52	***	(1.47, 1.58)	1.52	***	(1.47, 1.58)	1.52	***	(1.47, 1.59)	1.52	***	(1.47, 1.59)
$150,000 and above	2.11	***	(1.99, 2.24)	2.11	***	(1.99, 2.24)	2.11	***	(1.99, 2.24)	2.11	***	(1.99, 2.24)
**Work status (ref = no work)**												
Government	1.33	***	(1.28, 1.39)	1.33	***	(1.28, 1.39)	1.33	***	(1.28, 1.39)	1.33	***	(1.28, 1.39)
Private company	1.00		(0.98, 1.03)	1.00		(0.98, 1.03)	0.99		(0.97, 1.03)	0.99		(0.97, 1.02)
Non-profit	1.79	***	(1.71, 1.89)	1.79	***	(1.71, 1.89)	1.79	***	(1.71, 1.89)	1.79	***	(1.71, 1.89)
Others	0.66	***	(0.64, 0.69)	0.66	***	(0.64, 0.69)	0.66	***	(0.64, 0.69)	0.66	***	(0.64, 0.69)
**Health insurance (ref = public)**												
Private	1.35	***	(1.33, 1.38)	1.35	***	(1.32, 1.38)	1.35	***	(1.33, 1.39)	1.35	***	(1.33, 1.39)
Both	1.24	***	(1.23, 1.27)	1.24	***	(1.23, 1.27)	1.24	***	(1.23, 1.27)	1.24	***	(1.23, 1.27)
None	0.75	***	(0.73, 0.78)	0.75	***	(0.73, 0.78)	0.75	***	(0.73, 0.78)	0.75	***	(0.73, 0.78)
**Anxiety (ref = no)**												
Yes	1.23	***	(1.22, 1.26)	1.23	***	(1.22, 1.26)	1.23	***	(1.22, 1.26)	1.23	***	(1.21, 1.26)
**Depression (ref = no)**												
Yes	1.05	***	(1.03, 1.07)	1.05	***	(1.03, 1.07)	1.05	***	(1.03, 1.07)	1.05	***	(1.03, 1.07)
**Place of residence (ref = non-metro)**												
Metropolitan Area	1.32	***	(1.17, 1.5)	1.32	***	(1.17, 1.5)	1.32	***	(1.17, 1.5)	1.32	***	(1.17, 1.5)
**Survey week (ref = July 21–August 2, 2021)**												
August 4 – August 16	1.01		(0.98, 1.05)	1.01		(0.98, 1.05)	1.01		(0.98, 1.05)	1.01		(0.98, 1.05)
August 18 – August 30	1.09	**	(1.04, 1.15)	1.09	**	(1.04, 1.15)	1.09	**	(1.04, 1.15)	1.09	**	(1.04, 1.15)

Variables	Employment income loss × Gender identity			Housing instability × Gender identity			Food insufficiency × Gender identity			HH expense difficulty × Gender identity		
	OR		(95% CI)	OR		(95% CI)	OR		(95% CI)	OR		(95% CI)
September 1 - September 13	1.14	***	(1.09, 1.2)	1.14	***	(1.09, 1.2)	1.14	***	(1.09, 1.2)	1.14	***	(1.09, 1.2)
September 15 – September 27	1.12	***	(1.07, 1.19)	1.12	***	(1.07, 1.19)	1.12	***	(1.07, 1.19)	1.12	***	(1.07, 1.19)
September 29 - October 11	1.16	***	(1.1, 1.24)	1.16	***	(1.1, 1.24)	1.16	***	(1.1, 1.24)	1.16	***	(1.1, 1.24)
December 1 – December 13	1.30	***	(1.24, 1.39)	1.30	***	(1.24, 1.39)	1.30	***	(1.23, 1.39)	1.30	***	(1.23, 1.39)
December 29, 2021 – January 10, 2022	1.37	***	(1.3, 1.46)	1.37	***	(1.3, 1.46)	1.37	***	(1.3, 1.45)	1.37	***	(1.3, 1.45)
January 26 – February 7, 2022	1.36	***	(1.28, 1.45)	1.36	***	(1.28, 1.45)	1.36	***	(1.28, 1.45)	1.36	***	(1.28, 1.45)
March 2 – March 14	1.41	***	(1.34, 1.5)	1.41	***	(1.34, 1.5)	1.41	***	(1.34, 1.49)	1.41	***	(1.34, 1.49)
March 30 – April 11	1.28	***	(1.22, 1.36)	1.28	***	(1.22, 1.36)	1.28	***	(1.22, 1.36)	1.28	***	(1.22, 1.36)
April 27 – May 9	1.29	***	(1.23, 1.36)	1.29	***	(1.23, 1.36)	1.29	***	(1.23, 1.36)	1.29	***	(1.23, 1.36)
June 1 - June 13	1.27	***	(1.2, 1.36)	1.28	***	(1.2, 1.36)	1.27	***	(1.2, 1.36)	1.27	***	(1.2, 1.36)
June 29 - July 11	1.29	***	(1.24, 1.36)	1.29	***	(1.24, 1.36)	1.29	***	(1.23, 1.36)	1.29	***	(1.23, 1.36)
July 27 - August 8	1.17	***	(1.11, 1.25)	1.17	***	(1.11, 1.25)	1.17	***	(1.11, 1.25)	1.17	***	(1.11, 1.25)
September 14 - September 28	1.10	***	(1.05, 1.17)	1.10	***	(1.05, 1.17)	1.10	**	(1.05, 1.17)	1.10	**	(1.05, 1.17)
October 5 - October 17	1.07	*	(1.01, 1.14)	1.07	*	(1.01, 1.14)	1.07	*	(1.01, 1.14)	1.07	*	(1.01, 1.13)
November 2 - November 14	1.08	*	(1.02, 1.15)	1.08	*	(1.02, 1.15)	1.08	*	(1.02, 1.15)	1.08	*	(1.02, 1.15)
December 9 - December 19, 2022	1.13	***	(1.08, 1.2)	1.13	***	(1.08, 1.2)	1.13	***	(1.08, 1.2)	1.13	***	(1.08, 1.2)
January 4 - January 16, 2023	1.14	***	(1.09, 1.2)	1.14	***	(1.09, 1.2)	1.14	***	(1.09, 1.2)	1.14	***	(1.09, 1.2)
February 1 - February 13	1.13	***	(1.08, 1.2)	1.13	***	(1.08, 1.2)	1.13	***	(1.08, 1.2)	1.13	***	(1.08, 1.2)
March 1 - March 13	1.11	**	(1.05, 1.19)	1.11	**	(1.05, 1.19)	1.11	**	(1.05, 1.19)	1.11	**	(1.05, 1.19)
March 29 - April 10	1.11	***	(1.06, 1.18)	1.11	***	(1.06, 1.18)	1.11	***	(1.06, 1.18)	1.11	***	(1.06, 1.18)
April 26 - May 8	1.11	***	(1.05, 1.17)	1.11	***	(1.05, 1.17)	1.10	***	(1.05, 1.17)	1.10	***	(1.05, 1.17)
June 7 – June 19	1.12	***	(1.07, 1.19)	1.12	***	(1.07, 1.19)	1.12	***	(1.06, 1.19)	1.12	***	(1.06, 1.19)
June 28 – July 10	1.10	***	(1.05, 1.16)	1.10	***	(1.05, 1.16)	1.10	***	(1.05, 1.16)	1.10	***	(1.05, 1.16)
July 26 – August 7	1.09	**	(1.04, 1.15)	1.09	**	(1.04, 1.15)	1.09	**	(1.04, 1.15)	1.09	**	(1.04, 1.15)
August 23 – September 4	1.08	*	(1.02, 1.16)	1.08	*	(1.02, 1.16)	1.08	*	(1.02, 1.16)	1.08	*	(1.02, 1.16)
September 20 - October 2	1.10	**	(1.04, 1.18)	1.10	**	(1.04, 1.18)	1.10	**	(1.04, 1.17)	1.10	**	(1.04, 1.17)
October 18 - October 30, 2023	1.07	*	(1.02, 1.13)	1.07	*	(1.02, 1.13)	1.07	*	(1.02, 1.13)	1.07	*	(1.02, 1.13)
January 9 - February 5, 2024	0.93	*	(0.89, 1)	0.93	*	(0.89, 1)	0.93	*	(0.88, 1)	0.93	*	(0.88, 1)
February 6 - March 4	0.90	***	(0.85, 0.96)	0.90	***	(0.85, 0.96)	0.90	***	(0.85, 0.96)	0.90	***	(0.85, 0.96)
March 5 - April 1	0.91	**	(0.86, 0.96)	0.91	**	(0.86, 0.96)	0.91	***	(0.86, 0.96)	0.91	***	(0.86, 0.96)
April 2 - April 29	0.94	*	(0.89, 1)	0.94	*	(0.89, 1)	0.94	*	(0.89, 1)	0.94	*	(0.89, 1)
April 30 - May 27	0.92	**	(0.87, 0.98)	0.92	**	(0.87, 0.98)	0.92	**	(0.87, 0.98)	0.92	**	(0.87, 0.98)
May 28 - June 24	0.93	*	(0.89, 0.99)	0.93	*	(0.89, 0.99)	0.93	*	(0.89, 0.99)	0.93	*	(0.89, 0.99)
June 25 - July 22, 2024	0.93	**	(0.89, 0.98)	0.93	**	(0.89, 0.99)	0.93	**	(0.89, 0.98)	0.93	**	(0.89, 0.98)
**Constant**	1.85	***	(1.55, 2.21)	1.84	***	(1.55, 2.2)	1.88	***	(1.58, 2.25)	1.91	***	(1.6, 2.3)

Note: OR = Odds ratio. CI = Confidence interval. Ref = Reference group. A&PI = Asian and Pacific Islander. AA = Associate in Arts. BA+ = Bachelor's degree or higher. HH = Household. MSA = Metropolitan statistical area. Standard errors were clustered at the state level.
